# Mechanistic evaluation of surfactant assisted smart water flooding with sulfate and phosphate anions

**DOI:** 10.1038/s41598-025-17006-0

**Published:** 2025-08-27

**Authors:** Mona Zahedi, Amir Hossein Saeedi Dehaghani

**Affiliations:** Department of Petroleum Engineering, Faculty of Chemical Engineering, Tarbiat Modares University, P.O.BOX 14115-143, Tehran, Iran

**Keywords:** Enhanced oil recovery, Smart water, Cationic surfactant, Phosphate, Sulfate, Chemical engineering, Solid Earth sciences

## Abstract

Enhanced Oil Recovery (EOR) poses a significant challenge for carbonate rock reservoirs in the oil and gas industry. To address this, researchers have introduced methods such as smart water and surfactant-assisted injections. The complex and heterogeneous nature of these rock formations requires a comprehensive understanding of the processes during injection. In recent years, researchers have used phosphate and sulfate anions for smart water injections. While sulfate anion injections have received considerable attention, a significant gap remains in understanding the impact of phosphate anions. This gap calls for further exploration and study to uncover the potential effects of phosphate anion injections. A comprehensive understanding of their EOR mechanisms has yet to be efficiently achieved. This study aimed to examine the impact of sulfate (${\text{SO}}_{4}^{2-}$), mono- (${\text{HPO}}_{4}^{2-}$) and dihydrogen (${\text{H}}_{2}{\text{PO}}_{4}^{-}$) phosphate anions on altering wettability in oil-wet carbonate rock and reducing water/oil interfacial tension. The simultaneous use of these anions with a cationic surfactant, Cetyl Trimethyl Ammonium Bromide (CTAB) was also investigated. These mechanisms were explored through experimental tests, including contact angle measurements, fourier-transform infrared spectroscopy-attenuated total reflectance (FTIR-ATR) imaging, zeta potential measurements, and spontaneous imbibition. Contact angle measurements showed that ${\text{HPO}}_{4}^{2-}$ and ${\text{H}}_{2}{\text{PO}}_{4}^{-}$ anions perform better than ${\text{SO}}_{4}^{2-}$ in restoring water-wetness to carbonate rocks. FTIR-ATR analysis confirmed that carbonate rock exhibits higher water-wettability in solutions enriched with ${\text{H}}_{2}{\text{PO}}_{4}^{-}$. Zeta potential assessments showed a shift in the charge of oil-wet carbonate rock: from -54.9 mV to -32.4 mV with ${\text{SO}}_{4}^{2-}$, -16.4 mV with ${\text{H}}_{2}{\text{PO}}_{4}^{-}$, and -24.8 mV with ${\text{HPO}}_{4}^{2-}$. Moreover, the spontaneous imbibition test enabled us to calculate oil recovery. The computed oil recovery values for low-salinity water enriched with ${\text{H}}_{2}{\text{PO}}_{4}^{-}$, ${\text{HPO}}_{4}^{2-}$, and ${\text{SO}}_{4}^{2-}$ were 48%, 44%, and 36%, respectively. Finally, these recovery values significantly increased to 78%, 74%, and 66% for ${\text{H}}_{2}{\text{PO}}_{4}^{-}$, ${\text{HPO}}_{4}^{2-}$, and ${\text{SO}}_{4}^{2-}$ solutions after exposure of the core plug sample to CTAB. Therefore, the presence of ${\text{H}}_{2}{\text{PO}}_{4}^{-}$ ion, in conjunction with CTAB, yielded the most favorable results across all conducted tests.

## Introduction

Carbonate reservoirs contain over half of the world’s oil reserves^1^. Their distinct features include fractures, vugs, neutral to oil-wet wettability, low permeability, and a heterogeneous structure^2,3^. These unique characteristics necessitate a comprehensive understanding of these reservoirs^4,5^^.^ The carbonate rocks are oil-wet and naturally fractured^6^. Therefore, waterflooding performance in carbonate reservoirs is lower than in sandstone reservoirs^7–9^ Smart water flooding in both carbonate and sandstone rocks has received much attention. This method can increase the hydrocarbon recovery factor by almost 10% without any specific chemical additives. Moreover, it is cost-effective and environmentally friendly^10–12^.

Smart water injections include salinity water, modified water, designed water, nano-smart water, advanced ion management, and engineered water^13–19^. The primary goal of these injections is to improve oil recovery from reservoir rocks by altering wettability toward a more water-wet state^20–22^. Researchers have proposed various mechanisms for wettability alteration, including rock dissolution, ion displacement, and surface charge alteration^23–25^. The expansion of the double layer and differences in osmotic pressure also influence wettability alteration during low salinity water injection^26^. Nasralla et al.^27^ confirmed that rock dissolution did not occur during smart water injections. Moreover, some studies showed that in low salinity injections, ion displacement is the primary factor, with rock dissolution as a secondary contributor to wettability alteration^28–30^.

Optimizing the salinity and type of soluble ions in smart water can positively affect rock properties. Many studies have investigated the effects of water salinity and ion type. Some studies showed that smart water alters wettability towards a more water-wet state more effectively than formation water (FW). This is due to the presence of sulfate, calcium, and magnesium ions^26,31–35^. Increasing effective ion concentrations, particularly sulfate ions, to an optimal level yields better wettability alteration. This optimal ion concentration helps effective ions access the rock surface by minimizing non-active ions like Na^+^ and Cl^-^^31,36–39^. Karimi et al.^40^ found that sulfate-enriched diluted solutions were more effective in altering wettability and increasing oil recovery than sulfate-free samples. They explored various smart water formulations with sulfate and magnesium. In a similar research, Maghsoudian et al.^41^ examined the impact of varying concentrations of Ca^2+^, Mg^2+^, and ${\text{SO}}_{4}^{2-}$ on smart water performance. They concluded that sulfate-rich (SW2S), calcium-rich (SW4Ca), and magnesium-rich (SW2Mg) solutions yielded the best results. The sulfate ion notably enhances the capacity for carboxyl separation from carbonate rock surfaces^42,43^. Gandomkar and Rahimpour^44^ confirmed that a diluted solution rich in divalent cations and sulfate anions is the optimal smart water formulation.

Recent studies have explored the impact of surfactants on wettability alteration of sandstone^45,46^ and carbonate reservoirs^47–51^^.^ Aghdam et al.^46^ showed that different surfactants improved oil recovery in clay-rich sandstone by reducing IFT and altering wettability, with CTAB being the most effective due to its strong interaction with acidic oil components. However, investigating surfactant effects on carbonate reservoir rocks has garnered more interest due to the complexity of their structure. Jarrahian et al.^52^ investigated cationic, anionic, and non-ionic surfactants. They found that cationic surfactants had the most significant impact, while anionic surfactants had the least. Cationic surfactants were particularly effective in carbonate reservoirs. Kumar et al.^53^ studied various Trimethyl Ammonium Bromide (C_n_TAB) cationic surfactants on surface tension reduction and wettability alteration of carbonate rocks. They found that C_19_TAB was the most effective. Saien et al.^54^ investigated the combined effects of a benzimidazolium cationic GSAIL, and SDS surfactants on the interfacial tension (IFT), emulsification, and wettability alteration of the crude oil − water system. The results showed significant synergies, with up to 97.6% reduction in IFT. Researchers have also focused on combining surfactants and smart water as a new EOR method. Moradi et al.^55^ reported that smart water with a natural plant surfactant and different concentrations of active ions changed rock wettability and increased oil recovery. Mohammadi et al.^56^ observed similar results with the anionic surfactant (SDS) and low salinity water, although the sulfate effect disappeared in the presence of SDS. Other studies reported the highest oil recovery from carbonate rock when smart water contained the optimal concentration of active ions and lacked non-active ions^47^. Shahbazi et al.^57^ demonstrated the effects of a modified sulfate brine solution and a cationic surfactant on wettability alteration and oil recovery. They used contact angle and spontaneous imbibition tests. Their results highlighted the positive influence of surfactants in enhancing oil recovery.

A few studies have examined the impact of other water-soluble multivalent anions, such as phosphate and borate, on enhancing smart water performance with and without surfactants. These anions are often present in surface water compositions. Gupta et al.^58^ used two distinct smart water solutions derived from seawater (SW), substituting phosphate and borate anions for sulfate anions. Core flood tests with these solutions showed oil recovery increases of 20%, 16%, and 5% for phosphate, borate, and optimal sulfate concentrations, respectively. However, the exact mechanism behind this increase remained unclear. Meng et al.^59^ explored the impact of varying phosphate concentrations within different SW dilutions. They found a positive correlation between increased phosphate concentration and both wettability alteration and surface tension reduction. They concluded that phosphate concentration had a more pronounced influence than sulfate anions. Notably, these studies did not specify the types of salts used, which could lead to divergent results.

This study aims to investigate the mechanisms underlying wettability alteration in oil-wet carbonate rock using mono- (${\text{HPO}}_{4}^{2-}$) and dihydrogen (${\text{H}}_{2}{\text{PO}}_{4}^{-}$) phosphate anions. It also examines the interplay of surface tensions between water and oil. The investigation focuses on two primary objectives. First, it compares the impact of smart water solutions enriched with ${\text{HPO}}_{4}^{2-}$ and ${\text{H}}_{2}{\text{PO}}_{4}^{-}$ against smart water containing sulfate (${\text{SO}}_{4}^{2-}$) anions. Second, it explores the combined effect of smart water enriched with specific anions and Cetyl Trimethyl Ammonium Bromide (CTAB), a cationic surfactant. To accomplish these goals, a series of related experimental tests are considered. These tests include contact angle measurements, Fourier Transform Infrared (FTIR) spectroscopy of oil-wet thin sections treated with smart water, and zeta potential tests for wettability analysis. Additionally, the study assesses IFT and performs spontaneous imbibition tests to evaluate the interactions between oil and smart water surface tensions.

## Materials and experimental procedures

### Materials

#### Oil phase

An oil sample was provided from an Iranian oil field. The properties of the oil sample are listed in Table 1.

**Table 1:** Physical properties and compositional characteristics of the crude oil sample used in all experiments, including API gravity, viscosity, and SARA (saturates, aromatics, resins, and asphaltenes) fractions.

Property	Unit	Result
Gravity	API	34
Asphaltene	wt.%	5.72
Resin	wt.%	18.18
Aromatics	wt.%	60.19
Saturated	wt.%	15.91
Viscosity	centipoise	6.31

#### Rock sample

This study used carbonate rock samples of uniform size. Polished and flat thin sections were extracted, each two mm thick and 20 × 20 mm^2^ surface area, for the contact angle measurement and FTIR tests. Moreover, rock powders were used for the zeta potential measurement. Imbibition tests were conducted on three similar core samples, whose properties are shown in Table 2. In addition, Table 3 details the X-ray fluorescence (XRF) analysis of the carbonate rock sample. Loss on Ignition (LOI) value of 42.57% is reported to assess the purity of the carbonate (calcite) rock. This value is consistent with the expected weight loss from the thermal decomposition of CaCO₃, during which approximately 44% of the mass is released as CO₂. The result suggests a high-purity sample, estimated at around 98%. Reporting the LOI also helps to clarify the rock’s mineralogical composition and provides insight into its surface behavior when in contact with ionic solutions and surfactants. Moreover, the purity of rock samples indicates uniform mineralogy. The variation in density of core plugs arises from microporosity, microfractures, or minor measurement errors common in natural samples. Despite this, permeability values (800 mD) are consistent across all plugs, confirming that the density difference does not significantly affect the analysis.

**Table 2:** Properties of core samples.

Core number	Diameter (cm)	Length (cm)	Dry mass (gr)	Saturated mass (gr)	Porosity (%)	Permeability (mD)
1	3.8	5	81.34	100.7	0.33	800
2	3.8	5	81.77	100.2	0.31	800
3	3.8	5.2	88.53	107.6	0.33	800

**Table 3:** The XRF result of carbonate rock.

Composition	wt.%	Composition	wt.%
Loss on ignition	42.57	SO_3_	0.161
Na_2_O	0.045	Cl	0.034
MgO	0.382	CaO	56.25
Al_2_O_3_	0.114	Fe_2_O_3_	0.077
SiO_2_	0.223	Zn	0.108
P_2_O_5_	0.005	Sr	0.024

#### Aqueous phase

Synthetic brines were prepared by dissolving pure salts from the Merck Company in precise quantities of distilled deionized water (DIW). Table 4 details the properties of these salts. All solubility measurements reported were conducted at 25 °C. 15 distinct brine solutions were formulated for this research. Table 5 provides details about the types and concentrations of ions in these solutions. Additionally, a solution was used by diluting SW 10 times (10d). The SW formulation used in this work was based on the measured composition of Persian Gulf water. In this study, the 10d contains 0.00347 g/mole molar concentration of ${\text{SO}}_{4}^{2-}$. This molar concentration value of the ${\text{SO}}_{4}^{2-}$ in the 10d is denoted as 'm' and used for preparing smart waters. To produced smart waters, first this amount of ${\text{SO}}_{4}^{2-}$ (m) was omitted from the 10d (10d0S) to have better comparison between anions. Then, smart waters were produced in various molar concentrations of ${\text{H}}_{2}{\text{PO}}_{4}^{-}$, ${\text{HPO}}_{4}^{2-}$, and ${\text{SO}}_{4}^{2-}$ anions into 10d0S. The molar concentrations were set at 1/3, 1, 2, 4, and 8 times of m. Moreover, CTAB, a cationic surfactant, was used in this study. Table 6 shows its properties. CTAB was incorporated at a concentration of 0.364 wt% (equal to 1 CMC in 10d0S) into the smart water solution. Then, the combined effect of CTAB was examined with ${\text{H}}_{2}{\text{PO}}_{4}^{-}$, ${\text{HPO}}_{4}^{2-}$ and ${\text{SO}}_{4}^{2-}$ anions.

**Table 4:** Chemical properties of salts used in smart water preparation, including their molecular weights and water solubility at 25 °C.

Salt	Chemical formula	Molecular mass (g/mol)	Solubility in water (g/100cc)
Sodium chloride	NaCl	58.44	35.89
Calcium chloride	CaCl_2_	110.99	74.05
Potassium chloride	KCl	74.55	34.02
Magnesium chloride hexahydrate	MgCl_2_.6H_2_O	203.31	54.06
Sodium sulfate	Na_2_SO_4_	142.04	19.50
Sodium dihydrogen phosphate	NaH_2_PO_4_	119.98	86.9
Disodium hydrogen phosphate	Na_2_HPO_4_	141.93	11.8

**Table 5:** Ionic composition, pH, and total dissolved solids (TDS) of the 15 smart water formulations used in the experiments. The table presents the concentrations of key ions, including ${\text{SO}}_{4}^{2-}$, ${\text{H}}_{2}{\text{PO}}_{4}^{-}$, and ${\text{HPO}}_{4}^{2-}$, at various molar ratios (X = 1/3, 1, 2, 4, and 8) relative to the baseline sulfate concentration (m) in diluted SW (10d0S).

**Solutions**	${\mathbf{N}\mathbf{a}}^{2}$	${\mathbf{C}\mathbf{l}}^{-}$	${\mathbf{K}}^{+}$	${\mathbf{M}\mathbf{g}}^{2+}$	${\mathbf{C}\mathbf{a}}^{2+}$	$\mathbf{S}{\mathbf{O}}_{4}^{2-}$	$\mathbf{H}\mathbf{P}{\mathbf{O}}_{4}^{2-}$	${\mathbf{H}}_{2}\mathbf{P}{\mathbf{O}}_{4}^{-}$	**TDS**	**pH**
	ppm									
$\text{FW}$	44,346	77,676	2,086	797	5,286	667	0	0	130,858	6.6
$\text{SW}$	12,882	23,738	537	1,751	472	3,339	0	0	42,719	6.8
$10\text{d}0\text{S}$	1,128	2,374	53.6	1,752	472	0	0	0	5,780	6.7
$10\text{d}0\text{S}+1/3{\text{mH}}_{2}{\text{PO}}_{4}^{-}$	1,155	2,374	53.6	1,752	472	0	0	112	5,919	5
$10\text{d}0\text{S}+{\text{mH}}_{2}{\text{PO}}_{4}^{-}$	1,208	2,374	53.6	1,752	472	0	0	337	6,197	4.7
$10\text{d}0\text{S}+2{\text{mH}}_{2}{\text{PO}}_{4}^{-}$	1,288	2,374	53.6	1,752	472	0	0	674	6,614	4.6
$10\text{d}0\text{S}+4{\text{mH}}_{2}{\text{PO}}_{4}^{-}$	1,448	2,374	53.6	1,752	472	0	0	1,348	7,448	4.5
$10\text{d}0\text{S}+8{\text{mH}}_{2}{\text{PO}}_{4}^{-}$	1,768	2,374	53.6	1,752	472	0	0	2,696	9,116	4.4
$10\text{d}0\text{S}+1/3{\text{mHPO}}_{4}^{2-}$	1,185	2,374	53.6	1,752	472	0	111	0	5,948	6.7
$10\text{d}0\text{S}+{\text{mHPO}}_{4}^{2-}$	1,288	2,374	53.6	1,752	472	0	333	0	4,272	6.8
$10\text{d}0\text{S}+2{\text{mHPO}}_{4}^{2-}$	1,448	2,374	53.6	1,752	472	0	667	0	6,768	6.8
$10\text{d}0\text{S}+4{\text{mHPO}}_{4}^{2-}$	1,768	2,374	53.6	1,752	472	0	1,334	0	7,755	6.9
$10\text{d}0\text{S}+8{\text{mHPO}}_{4}^{2-}$	2,408	2,374	53.6	1,752	472	0	2,668	0	9,730	7.1
$10\text{d}0\text{S}+1/3{\text{mSO}}_{4}^{2-}$	1,185	2,374	53.6	1,752	472	111	0	0	5,948	6.7
$10\text{d}0\text{S}+{\text{mSO}}_{4}^{2-}$	1,288	2,374	53.6	175	47.2	334	0	0	4,272	6.8
$10\text{d}0\text{S}+2{\text{mSO}}_{4}^{2-}$	1,448	2,374	53.6	1,752	472	668	0	0	6,768	6.8
$10\text{d}0\text{S}+4{\text{mSO}}_{4}^{2-}$	1,768	2,374	53.6	1,752	472	1,335	0	0	7,755	6.9
$10\text{d}0\text{S}+8{\text{mSO}}_{4}^{2-}$	2,408	2,374	53.6	1,752	472	2,671	0	0	9,730	7.1

**Table 6:** CTAB properties.

Chemical name	Unit	Molecular weight (g/mol)	Critical micelle concentration (wt. %)
CTAB or C_19_TAB	CH_3_(CH_2_)_15_N(Br)(CH_3_)_3_	364.46	0.364

### Experiments

#### Aging rock samples

Initially, the thin sections were placed into a Soxhlet apparatus and thoroughly washed them with toluene and methanol. Then, the washed thin sections were dried overnight in an oven. After drying, the thin sections were immersed in formation brine for one day and then in oil at 90°C for 8 weeks. This aging process prepared the thin sections for further testing. The thin sections were then ready for contact angle and Fourier-transform infrared spectroscopy-attenuated total reflectance (FTIR-ATR) tests. For zeta potential tests, oil-wet powder was needed. 1 gr of rock powder was dispersed in 10 ml of formation brine and let it stand for one day at 90°C. Then, 10 ml of oil was added to the mixture and stirred at 90°C for 48 h. The resulting mixture was separated using a centrifuge at 5300 rpm for 30 min. The obtained oil-wet powder was dried overnight at 40°C^52,60–63^.

#### Contact angle measurement

**Fig. 1:**
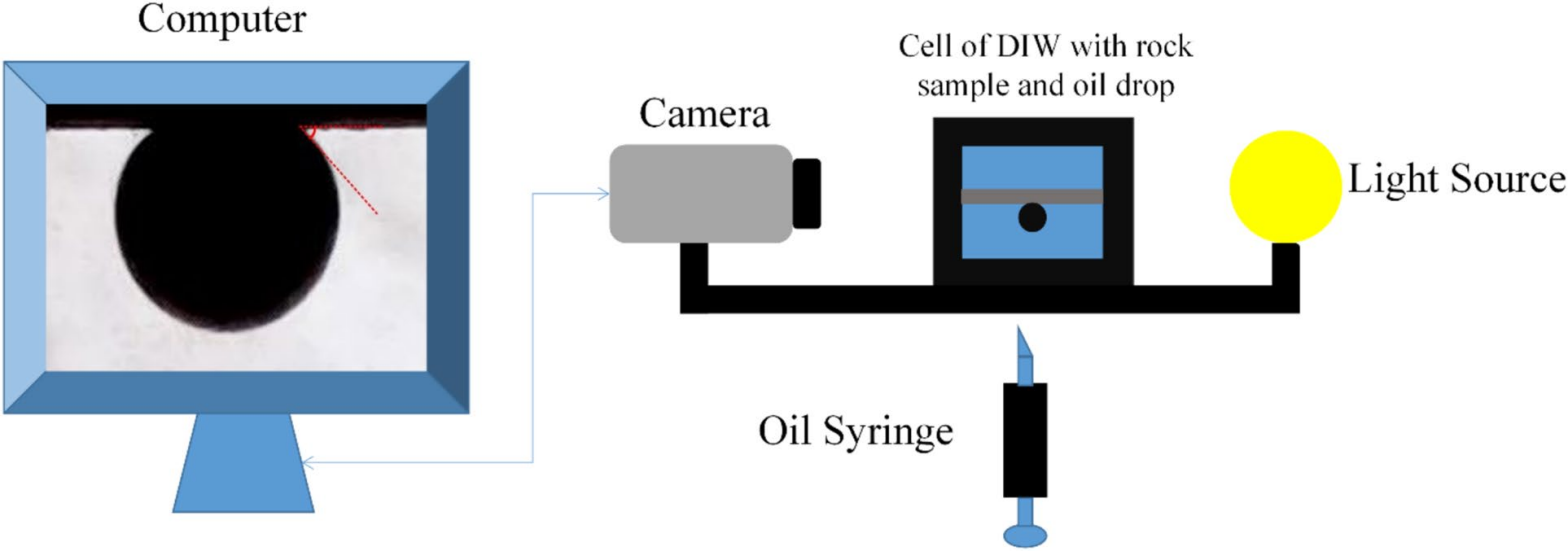
Schematic of the contact angle (and IFT) measuring setup.

The sessile drop method enabled direct measurement of contact angles for water-wet and oil-wet rock thin sections. Figure 1 shows how this technique assesses the wettability of the oil/water/rock system. First, the thin section was placed in a cell containing DIW. Next, a syringe was used to release an oil drop onto the rock’s surface from the bottom of the cell. Finally, an image of the oil drop was captured with a high-quality camera. The first step involves finding a solution with an optimal ion concentration to transform the wettability towards a water-wet state. 10d0S solution was selected to create different smart waters enriched with different molar concentrations of ${\text{H}}_{2}{\text{PO}}_{4}^{-}$, ${\text{HPO}}_{4}^{2-}$ and ${\text{SO}}_{4}^{2-}$.

#### Fourier Transform Infrared (FTIR)

FTIR is a valuable analytical technique that provides insights into the chemical bonds within organic and inorganic groups. FTIR spectra helps discern adsorbed functional groups on rock surfaces. In wettability alteration, the most effective smart water efficiently removes oil from the rock surface. Previous research highlighted the role of polar organic compounds, such as carboxylic acids, in altering the wettability of carbonate rocks towards an oil-wet state^60,61,64–66^. FTIR-ATR tests were used to identify organic functional groups absorbed on the surface of oil-wet carbonate rock before and after exposure to smart water^52,67^. The peak value indicated the amount of functional group adsorption on the surface^68^.

The emitted infrared spectrum captures oscillations from molecular vibrations and rotations. These oscillations characterize molecular structures, as each molecule and functional group have a distinct oscillation pattern. This technique investigates material adsorption at the interface of solids and liquids^69,70^. In this study, FTIR spectra was acquired using the PerkinElmer Spectrum, model 10.03.06. Oil functional groups were identified by preparing a solid tablet of 99 mg KBr powder and one mg reservoir oil (FTIR method). Moreover, a dried oil-wet thin section of carbonate rock, exposed to smart water, was positioned within the device (FTIR-ATR method). This enabled the analysis of oil adhering to the rock surface. After testing, a transmission intensity spectrum ranging from 0 to 100% was generated, plotted against wavelength from 400 cm⁻^1^ to 4000 cm⁻^1^.

#### Zeta potential

The zeta potential refers to the electric potential at the shear plane of a dispersed charged particle within a solution. This potential depends on the surface charge of these particles. In this study, the zeta potential was measured using PARTICLE METRIX. This specialized instrument quantifies the electrophoretic mobility of dispersed particles and then computes the zeta potential under ambient conditions using Henry’s equation (Eq. (1))^71^.

1 $${U}_{e}=\frac{2 \varepsilon \xi }{3\eta }f\left(ka\right)$$

where ${U}_{e}$ is the electrophoresis mobility, $\varepsilon$ is the permittivity, $\xi$ is zeta potential, $\eta$ is fluid viscosity, and $f\left(ka\right)$ is Henry’s equation.

The electrical characteristics of oil-wet calcite powder were investigated by assessing the zeta potential of the dispersed sample in smart water. The following protocol was followed to compute the zeta potential^60,61,72^: First, an initial solution containing 1 wt.% of oil-wet powder was prepared. Therefore, 0.2 gr of oil-wet rock powder into 20 ml of smart water was introduced. The mixture was vigorously shocked and placed in an oven at 90°C for one day. Next, the treated rock powder was separated from the smart water through centrifugation and dried overnight in an oven at 40°C. Finally, the treated rock powder was immersed in a 20 ml solution of DIW with a pH of 8, prepared using 0.01 M NaOH and 0.01 M HCl solutions.

#### IFT

**Fig. 2:**
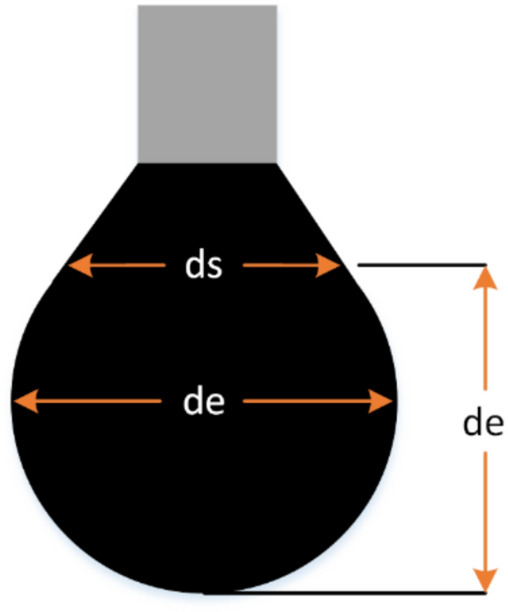
A drop of pendant oil from a needle.

The pendant drop method was used with the contact angle setup (shown in Fig. 1) to measure the IFT between oil and smart water. First, an oil drop was injected into a cell containing smart water. Before releasing the oil drop, an image was captured with a microscopic camera. Then, the IFT values were calculated using Eq. (2) in the MATLAB software.

2 $$\sigma =\frac{\Delta \rho g{d}_{e}^{2}}{H}$$

where $\sigma$ is the IFT, $\Delta \rho$ is the smart water and oil density difference, $g$ is gravity acceleration of earth, ${d}_{e}$ is the equatorial diameter of the drop, $H$ is a correction factor which is related to the shape factor ($S=\frac{{d}_{s} }{{d}_{e}}$), and as shown in Fig. 2, ${d}_{s}$ is drop diameter which is measured horizontally with a distance from the top of the drop.

#### Spontaneous imbibition

The spontaneous imbibition test determines oil recovery in reservoir rock samples, especially those with heterogeneity. In this study, the test was used to assess the impact of optimal solutions enriched with ${\text{SO}}_{4}^{2-}$, ${\text{HPO}}_{4}^{2-}$ and ${\text{H}}_{2}{\text{PO}}_{4}^{-}$ ions on oil production, both with and without CTAB. First, the plugs were thoroughly washed in a Soxhlet with methanol for 2 days and toluene for 14 days to remove organic and inorganic contaminants. Then, the plugs were dried at 90°C for 7 days. After drying, the plugs were fully saturated with FW for 7 days, followed by displacement of the brine with crude oil for 60 days. Then, the aged plugs were immersed in an Amott cell, exposing them to FW, SW, and formulated smart water. To extend the imbibition process, the plugs were exposed to CTAB while they were immersed in the smart water. The injection process continued for 60 days until oil production ceased. Finally, the amount of oil produced was documented.

#### Measurement accuracy and repeatability

To ensure accuracy and repeatability, all experiments were conducted at least three times, and the reported values represent the average of independent measurements. The contact angle device had a precision of ± 0.01°, and zeta potential measurements were performed under controlled temperature and pH conditions using identical powder samples, with observed variations within ± 2.5 mV. IFT tests were conducted using the pendant drop method and analyzed via a MATLAB code, with a computational error estimated below ± 0.2 mN/m. Additionally, spontaneous imbibition experiments were performed on three core plugs with similar porosity and permeability to minimize the effect of rock heterogeneity.

## Result and discussion

### Contact angle measurement

**Fig. 3:**
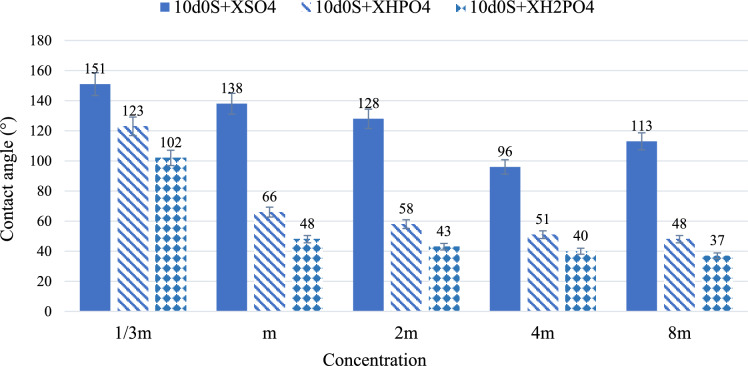
The results of wettability alteration for different smart waters in different concentration (X) of anions in absence of CTAB.

Figure 3 shows the results of wettability alteration for different smart waters in absence of CTAB. The initial contact angle of water-wet rock thin sections is 28°, increasing to 160° after aging. First, the contact angle of 10d0S brine is measured. This brine reduces the contact angle from 160° to 122°. Then, to evaluate the effect of ${\text{H}}_{2}{\text{PO}}_{4}^{-}$, ${\text{HPO}}_{4}^{2-}$ and ${\text{SO}}_{4}^{2-}$ on wettability alteration, the 10d0S solution is used with these anions at the same molar concentration. Moreover, the effect of different concentrations is investigated on wettability alteration in presence and absence of CTAB. These concentrations are 1/3, 1, 2, 4, and 8 times the molar concentration of sulfate anion in 10d, labeled as m. The results indicate that ${\text{H}}_{2}{\text{PO}}_{4}^{-}$ and ${\text{HPO}}_{4}^{2-}$ anions reduce the contact angle more than other anions and therefore perform better in wettability alteration to a water-wet state in absence of CTAB.

**Fig. 4:**
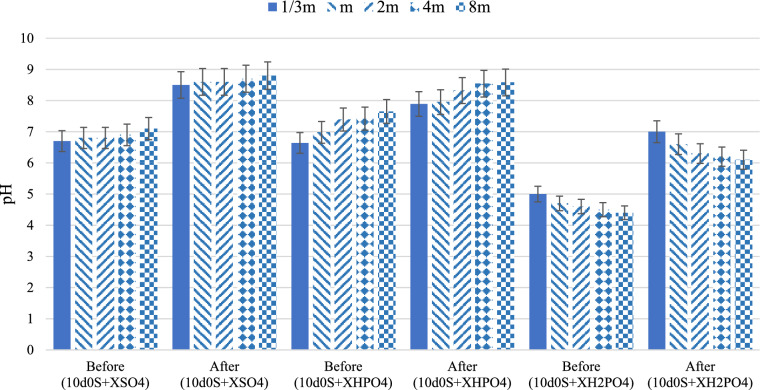
The pH of smart waters before and after exposure to the oil-wet carbonate rock thin sections in different concentration (X) of anions.

The results show that exposing oil-wet carbonate rock to smart water with varying amounts of ${\text{H}}_{2}{\text{PO}}_{4}^{-}$ decreases the contact angle between the oil drop and the rock surface. This decrease is more significant as the concentration of ${\text{H}}_{2}{\text{PO}}_{4}^{-}$ increases (from 1/3 m to 8 m), making the rock surface more water-wet (from 102° to 37°). Additionally, the results indicate that ${\text{H}}_{2}{\text{PO}}_{4}^{-}$ performs better than the other two anions (${\text{HPO}}_{4}^{2-}$ and ${\text{SO}}_{4}^{2-}$) in reducing the contact angle. The reason is dissolving ${\text{NaH}}_{2}{\text{PO}}_{4}$ salt in water produces ${\text{H}}_{2}{\text{PO}}_{4}^{-}$ anions (Eq. (3)) and then there also be H⁺ and ${\text{HPO}}_{4}^{2-}$ present in the water (Eq. (4)). Moreover, Fig. 4 shows that the initial pH values of solutions containing this salt are acidic, ranging from 5 to 4.4 as the concentration increases from 1/3 m to 8 m. As the salt concentration in water increases, the pH decreases. This confirms the presence of H⁺ ions and their increasing concentration as the ${\text{H}}_{2}{\text{PO}}_{4}^{-}$ concentration in smart water rises.

3 $${\left({NaH}_{2}P{O}_{4}\right)}_{\left(aq\right)}\stackrel{}{\leftrightarrow }{Na}^{+}+{H}_{2}P{O}_{4}^{-}$$

4 $${\left({H}_{2}P{O}_{4}^{-}\right)}_{\left(aq\right)}\stackrel{}{\leftrightarrow }HP{O}_{4}^{2-}+{H}^{+}$$

The presence of H⁺ ions alongside ${\text{HPO}}_{4}^{2-}$ and ${\text{H}}_{2}{\text{PO}}_{4}^{-}$ anions helps reduce the contact angle. ${\text{HPO}}_{4}^{2-}$ and ${\text{H}}_{2}{\text{PO}}_{4}^{-}$ anions approach the carbonate rock surface, increasing the negative charge potential around the rock. Consequently, the negatively charged carboxyls on the rock surface move away by forming complexes with active cations in smart water, such as Ca^2^⁺ and Mg^2^⁺. H⁺ in the smart water solution also competes with Ca^2^⁺ and Mg^2^⁺ cations to reach the carbonate rock surface and form complexes with carboxyl. H⁺ is absorbed by carboxyl (R-COO⁻) on the rock surface, forming carboxylic acid (R-COOH), which is soluble in water. Additionally, H⁺ in the smart water solution causes carbonates to react and separate from the rock surface, forming OH⁻ anions and CO₂ gas (Eq. (7)). The presence of OH⁻ in the aqueous solution reduces the concentration of H⁺ due to their tendency to react. As a result, more ${\text{H}}_{2}{\text{PO}}_{4}^{-}$ anions convert into ${\text{HPO}}_{4}^{2-}$ and H⁺ (Eq. (4)).

Therefore, the presence of H⁺ leads to the dissolution of carbonate rock, releasing Ca^2^⁺ into the water (Eq. (5)). As Ca^2^⁺ separate from the rock surface, the attached carboxyls also move away, making the rock water-wet. The pH of the smart water increases after exposure to the rock, confirming the dissolution of the carbonate rock and the release of OH⁻^73^. Contact angle results show that increasing ${\text{H}}_{2}{\text{PO}}_{4}^{-}$ concentration (from 1/3 m to 8 m) in smart water enhances wettability alteration (from 102° to 37°). These alterations are more significant compared to those caused by ${\text{SO}}_{4}^{2-}$ and ${\text{HPO}}_{4}^{2-}$ anions.

5 $$CaC{O}_{3}\stackrel{}{\leftrightarrow }{Ca}^{2+}+C{O}_{3}^{2-}$$

6 $$C{O}_{3}^{2-}+{H}^{+}\stackrel{}{\leftrightarrow }HC{O}_{3}^{-}$$

7 $$HC{O}_{3}^{-}\stackrel{}{\leftrightarrow }C{O}_{2}+O{H}^{-}$$

Smart waters containing ${\text{HPO}}_{4}^{2-}$ anion, as shown in Fig. 3, perform better than ${\text{SO}}_{4}^{2-}$ in reducing the contact angle (as the concentration increases from 1/3 m to 8 m, the ${\text{SO}}_{4}^{2-}$ decreases the contact angle from 151° to 113°, whereas the ${\text{HPO}}_{4}^{2-}$ decreases it from 123° to 48°). However, these solutions do not outperform those with ${\text{H}}_{2}{\text{PO}}_{4}^{-}$. Moreover, Fig. 4 shows increasing the concentration of this salt in water raises the pH (for 1/3 m, the pH increased from 6.6 to 7.9 and for 8 m, the pH increased from 7.6 to 8.6). This suggests that some ${\text{HPO}}_{4}^{2-}$ converts to ${\text{H}}_{2}{\text{PO}}_{4}^{-}$ by absorbing H⁺, and the presence of OH⁻ slightly increases the pH. Since the pH remains close to neutral, the most available anion in the water is ${\text{HPO}}_{4}^{2-}$. As mentioned, increasing the concentration of ${\text{HPO}}_{4}^{2-}$ in the smart water solution decreases the contact angle, altering wettability towards more water-wet. This indicates that higher anion concentration and their access to the carbonate rock surface increases the negative charge density. Consequently, more carboxyls on the surface form ion complexes with active cations like Ca^2^⁺ and Mg^2^⁺, moving away from the rock surface. The pH difference before and after exposing oil-wet carbonate rock to smart water with ${\text{HPO}}_{4}^{2-}$ shows active rock dissolution and ion movement (for 1/3 m, the pH increased from 6.6 to 7.9 and for 8 m, the pH increased from 7.6 to 8.6). Additionally, solutions containing ${\text{HPO}}_{4}^{2-}$ have a higher negative charge than those with ${\text{H}}_{2}{\text{PO}}_{4}^{-}$. However, the presence of H⁺, which interacts with both ${\text{HPO}}_{4}^{2-}$ and ${\text{H}}_{2}{\text{PO}}_{4}^{-}$, makes smart water with ${\text{H}}_{2}{\text{PO}}_{4}^{-}$ more effective than with ${\text{HPO}}_{4}^{2-}$.

8 $${Na}_{2}HP{O}_{4}\stackrel{}{\leftrightarrow }{2Na}^{+}+HP{O}_{4}^{2-}$$

9 $$HP{O}_{4}^{2-}+{H}_{2}O\stackrel{}{\leftrightarrow }{H}_{2}P{O}_{4}^{-}+O{H}^{-}$$

In this study, solutions containing ${\text{SO}}_{4}^{2-}$ among all smart waters have less effect on reducing the contact angle (as the concentration increases from 1/3 m to 8 m, the ${\text{SO}}_{4}^{2-}$ decreases the contact angle from 151° to 113°). The ${\text{SO}}_{4}^{2-}$ in smart water solutions acts through ion displacement, similar to the ${\text{HPO}}_{4}^{2-}$ anion. As the ${\text{SO}}_{4}^{2-}$ reaches the carbonate rock surface, it increases the negative charge around the rock. Consequently, negatively charged carboxyls loosen their bond with Ca^2^⁺ on the rock surface. These carboxyls then form ionic complexes with other cations in the water, such as Ca^2^⁺ and Mg^2^⁺, moving away from the rock surface. This process decreases the contact angle, making the rock surface more water-wet. As the concentration of ${\text{SO}}_{4}^{2-}$ increases, the contact angle reduction also increases because more carboxyls separate from the surface due to the higher negative charge density created by ${\text{SO}}_{4}^{2-}$. However, at 8 m concentration, further increasing the ${\text{SO}}_{4}^{2-}$ amount does not significantly affect the contact angle reduction. Excessive ${\text{SO}}_{4}^{2-}$ disrupts the ion movement process^74^. At 4 m, the contact angle decreases to 96°, whereas at 8 m, it increases to 113°. Comparing the pH levels before and after exposing carbonate rock to smart water containing ${\text{SO}}_{4}^{2-}$ indicates a dissolution mechanism of the carbonate rock (for 1/3 m, the pH increased from 6.7 to 8.5 and for 8 m, the pH increased from 7.1 to 8.8). Despite having the same size and ionic charge density, ${\text{SO}}_{4}^{2-}$ and ${\text{HPO}}_{4}^{2-}$ anions differ in their effectiveness. The results show that ${\text{HPO}}_{4}^{2-}$ is more effective in the ion displacement mechanism.

**Fig. 5:**
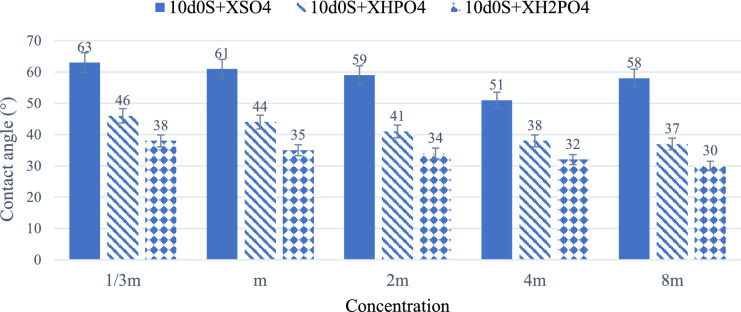
The results of the wettability alteration of different smart waters in different concentration (X) of anions in presence of CTAB.

As shown in Fig. 5, CTAB makes solutions containing ${\text{SO}}_{4}^{2-}$, ${\text{HPO}}_{4}^{2-}$ and ${\text{H}}_{2}{\text{PO}}_{4}^{-}$ ions more water-wet. As the concentration increases from 1/3m to 8m, the ${\text{SO}}_{4}^{2-}$ decreases the contact angle from 63° to 58°, the ${\text{HPO}}_{4}^{2-}$ decreases it from 46° to 37°, and the ${\text{H}}_{2}{\text{PO}}_{4}^{-}$ decreases it from 38° to 30°. CTAB in smart water solutions alters wettability more effectively than DIW enriched with 0.364 wt.% CTAB. Additionally, CTAB performs better in the presence of anions due to ion displacement^40^. CTAB has a hydrocarbon chain with a hydrophilic tail, known as a monomer. Negatively charged carboxyls attach to the carbonate rock surface, and anions in the stern layer increase the negative charge density. As a result, positively charged CTAB monomers move closer to the rock surface. These oil-wet monomers resemble carboxyl in structure and remove carboxyl from the rock surface by attaching to its negative tail.

**Fig. 6:**
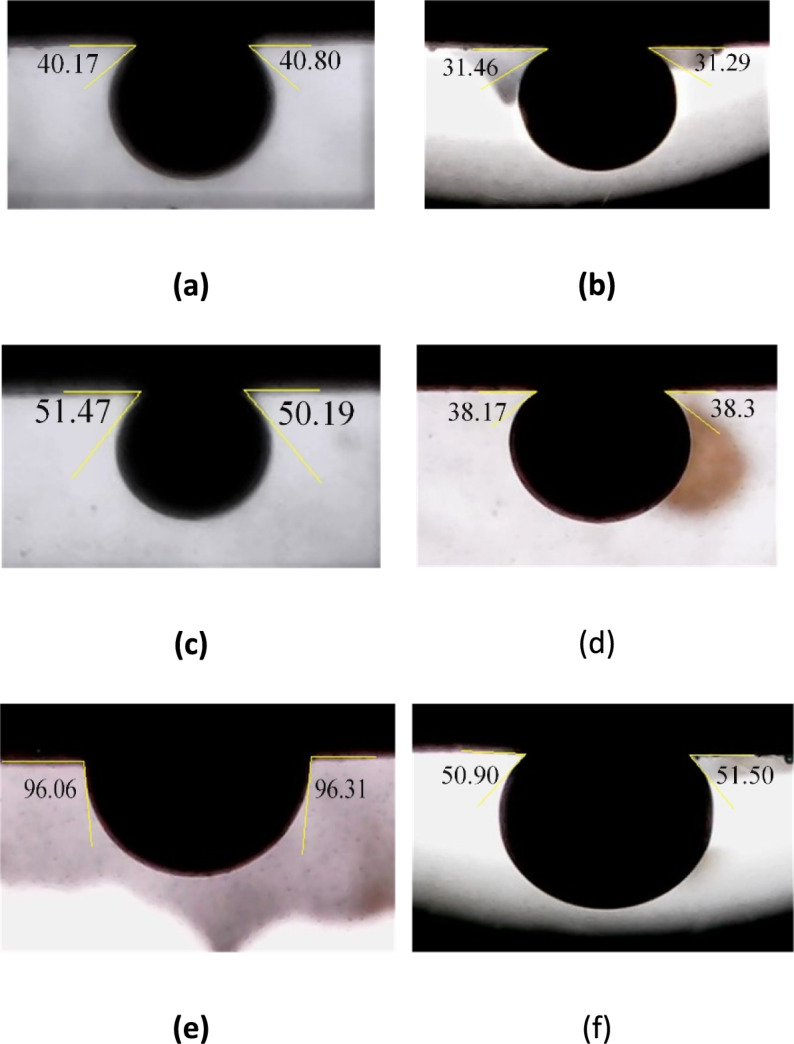
The final contact angle images of oil drops on the rock sections treated by (**a**) $10\text{d}0\text{S}+4{\text{mH}}_{2}{\text{PO}}_{4}^{-}$ ; (**b**) $10\text{d}0\text{S}+4{\text{mH}}_{2}{\text{PO}}_{4}^{-}$+CTAB ; (**c**) $10\text{d}0\text{S}+4{\text{mHPO}}_{4}^{2-}$ ; (**d**) $10\text{d}0\text{S}+4{\text{mHPO}}_{4}^{2-}$+CTAB ; (**e**) $10\text{d}0\text{S}+4{\text{mSO}}_{4}^{2-}$ ; (**f**)$10\text{d}0\text{S}+4{\text{mSO}}_{4}^{2-}$+CTAB smart waters in 4m anions concentrations in the presence and absence of CTAB.

The effect of solutions enriched by 4 m times ${\text{SO}}_{4}^{2-}$, ${\text{HPO}}_{4}^{2-}$ and ${\text{H}}_{2}{\text{PO}}_{4}^{-}$ anions concentrations were investigated in presence and absence of CTAB for our next experiments. Contact angle reduction peaks at 4 m, with no further improvement at higher concentrations, such as 8 m, due to surface saturation and salt precipitation. For phosphate ions, 4 m achieves a balance between surface charge enhancement and pH-driven desorption of acidic surface groups. Moreover, practical considerations such as solution stability further support this choice, as precipitation is observed at 8 m in some formulations. Therefore, the 8 m ${\text{HPO}}_{4}^{2-}$ solution forms a precipitate, and for ${\text{H}}_{2}{\text{PO}}_{4}^{-}$, there is only a slight difference in contact angle between the 4 m and 8 m solutions. Moreover, Fig. 6 shows the typical images of the oil drops on the rock sections treated by different smart waters in 4m times ${\text{SO}}_{4}^{2-}$, ${\text{HPO}}_{4}^{2-}$ and ${\text{H}}_{2}{\text{PO}}_{4}^{-}$ anions concentrates in the presence and absence of CTAB. Considering that the samples were exposed to crude oil for eight weeks before the experiments and were artificially made oil-wet (with an average initial contact angle of 160°), the substantial reduction in contact angle observed in these figures indicates a shift in wettability toward a water-wet state. This transition is attributed to physicochemical processes such as ion exchange, surface dissolution, and surfactant adsorption.

### Fourier Transform Infrared (FTIR)

**Fig. 7:**
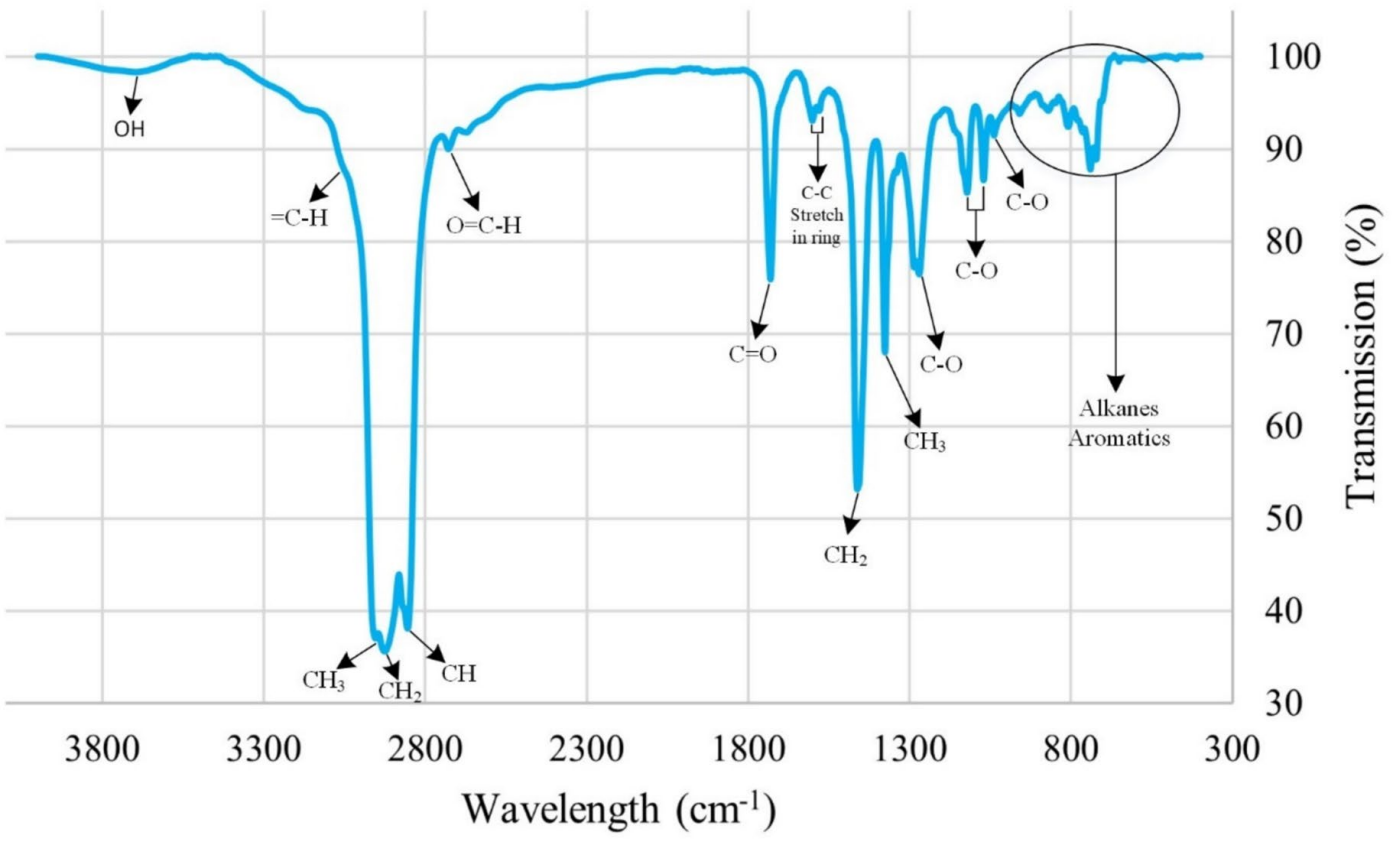
The result of the oil FTIR test.

Figure 7 shows the oil FTIR spectrum. The broad peak near 3000 cm^-1^ indicates the stretching vibration of the polar O–H bond in carboxylic compounds, which adhere to the positive surface of carbonate rock^75^. The peak at 1730 cm^-1^ represents the C = O stretch vibration in carboxylic acids. The peak at 3063 cm^-1^ relates to the C-H stretch in aromatics. Peaks between 1580 cm^-1^ and 1600 cm^-1^ indicate C–C stretches in aromatic rings. The peak at 2729 cm^-1^ shows the C-H stretch in aldehydes.

Oil is a complex mix of organic compounds, so the peak at 1730 cm^-1^ overlaps with C = O stretches in carboxylic acids, aldehydes, and unsaturated esters. Peaks at 1287 cm^-1^, 1124 cm^-1^, 1071 cm^-1^, and 1038 cm^-1^ correspond to C-O stretches in carboxylic acids and other organic compounds like aromatics and esters. Peaks at 2954 cm^-1^, 2926 cm^-1^, and 2855 cm^-1^ indicate C-H stretches in alkanes, while peaks at 1462 cm^-1^ and 1377 cm^-1^ represent bending/scissoring and methyl rocking vibrations in alkanes. Peaks below 1000 cm^-1^ show C-H vibrations in aromatics and long-chain alkanes^53,67^.

**Fig. 8:**
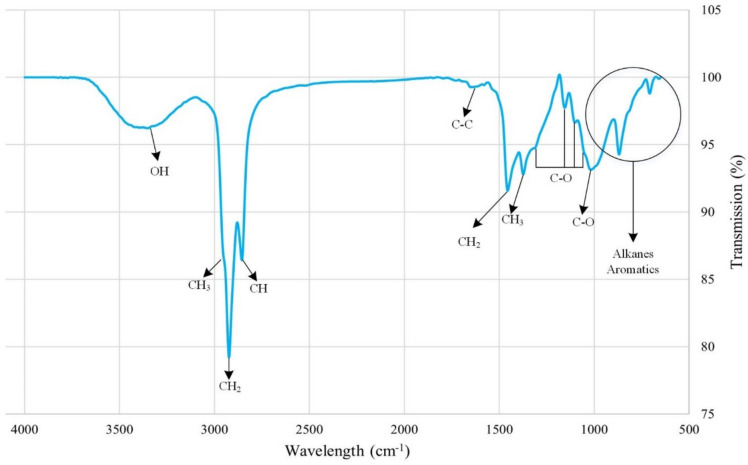
The FTIR-ATR test results on an oil-wet thin section.

Polar oil components, especially carboxylic acids, play a key role in altering wettability to oil-wet by adsorbing on carbonate rock surfaces. Figure 8 shows the FTIR-ATR spectra of carbonate rock after aging. It has a broad O–H band peak in carboxylic acids above 3000 cm^-1^. The peak at 1650 cm^-1^ shows the C–C stretch in aromatic rings. The peak at 3063 cm^-1^ is not visible, likely due to the weak presence of heavy polar compounds with higher carbon-to-hydrogen ratios. Peaks between 1000–1300 cm^-1^ indicate C-O stretches in polar organic compounds like carboxylic acids. Peaks below 1000 cm^-1^ indicate aromatic and alkane presence. The rest of the peaks relate to C-H vibrations in alkane chains. The FTIR-ATR results align with related research^53,67^.

**Fig. 9:**
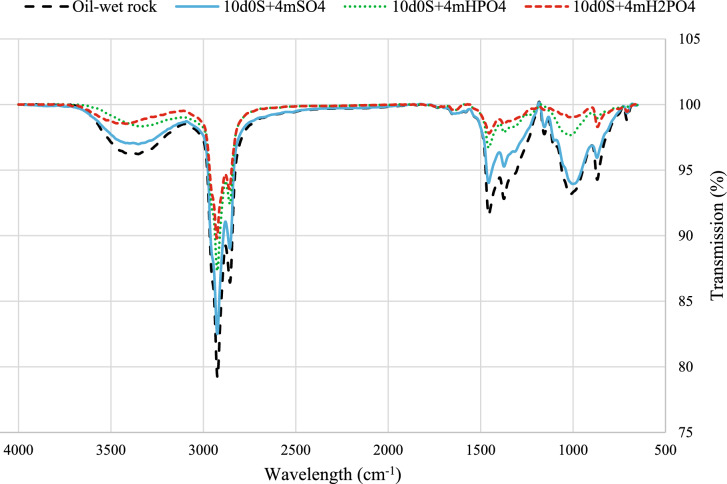
The FTIR-ATR test results of thin sections that were exposed to selected smart water.

Figure 9 shows FTIR-ATR spectra of thin sections exposed to smart waters. Deeper peaks indicate more oil on the rock surface, illustrating wettability alteration effects. In summary, the wettability alteration effect is as follows:

$${\text{H}}_{2}{\text{PO}}_{4}^{-}>{\text{HPO}}_{4}^{2-}>{\text{SO}}_{4}^{2-}$$

**Fig. 10:**
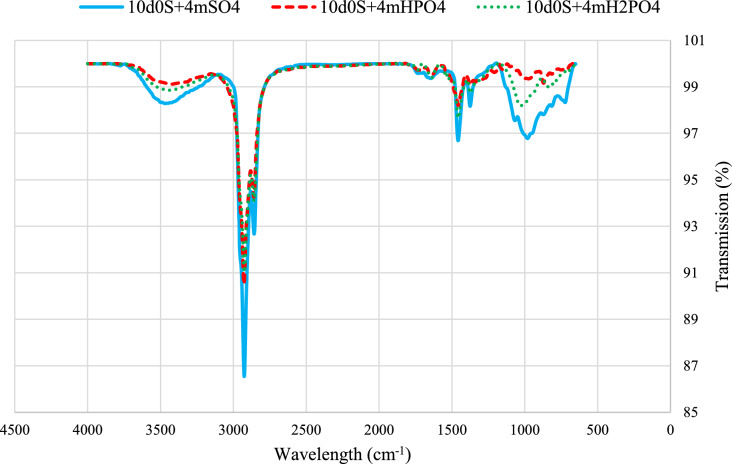
The FTIR-ATR test results of smart waters in presence of CTAB.

Figure 10 shows results for a thin section exposed to smart water with CTAB. The results agree with contact angle measurements, showing decreased peak intensities in the presence of the surfactant. This indicates enhanced removal of organic compounds by smart water. CTAB shows medium intensity peaks around 950 cm^-1^ and 750 cm^-1^, and stronger peaks at 1370 cm^-1^, 1460 cm^-1^, 2850 cm^-1^, and 2900 cm^-1^, indicating different resonances of C-H bonds in the alkane chain^53,68,76^.

**Fig. 11:**
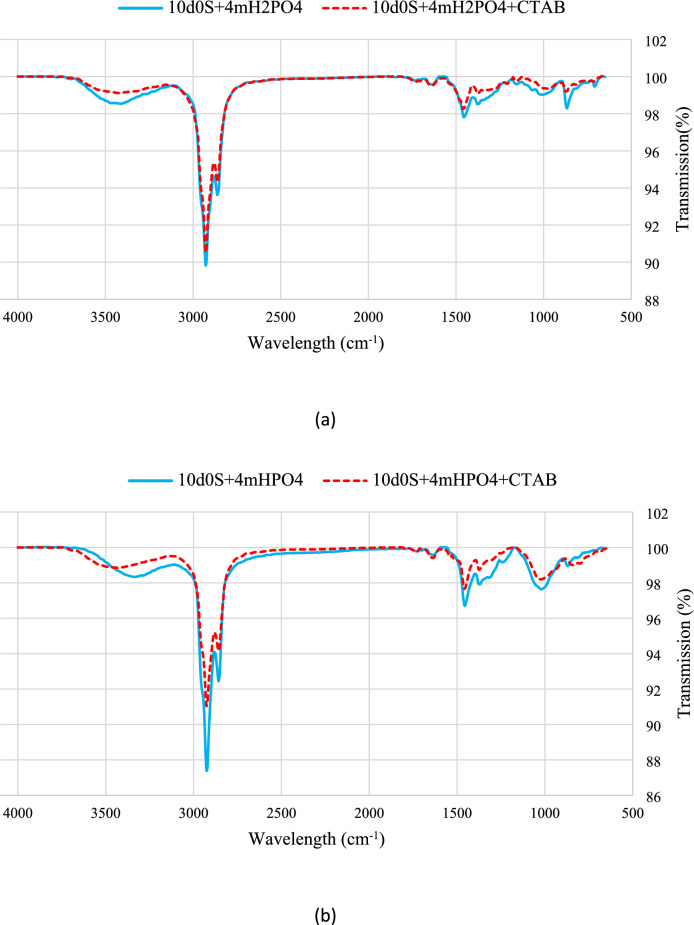

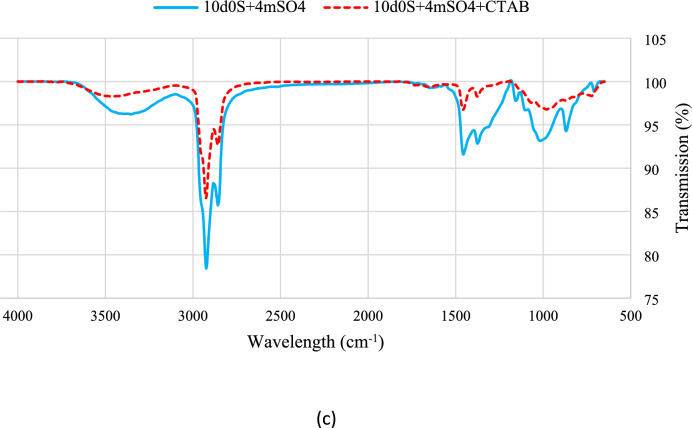
Comparison of the FTIR-ATR test spectrums for (**a**) 10d0S + 4mH2PO4. (**b**) 10d0S + 4mHPO4 (**c**) 10d0S + 4mSO4 smart waters in presence and absence of CTAB.

Figure 11 compares curves for smart water solutions with and without surfactant. A decrease in peak intensity shows the effectiveness of the surfactant.

### Zeta potential

The zeta potential of the carbonate rock surface is positive at a pH of less than 8 to 9.5^77^. In carbonate reservoirs, the water, rock, and oil system typically reach equilibrium after many years, resulting in a pH around 8. The zeta potential of oil-wet carbonate rock (composed of calcite) is more negative than that of water-wet carbonate rock. This is due to the negatively charged carboxyls, which accumulate on the surface of the positively charged carbonate rock, reducing the zeta potential at a pH below 8 to 9.5. Moreover, zeta potential depends on factors such as salinity, the type of anions, and the amount of carboxyls absorbed on the rock surface. Therefore, to focus on the effect of solutions on the change of zeta potential, factors such as pH and temperature were constant during measurement.

**Fig. 12:**
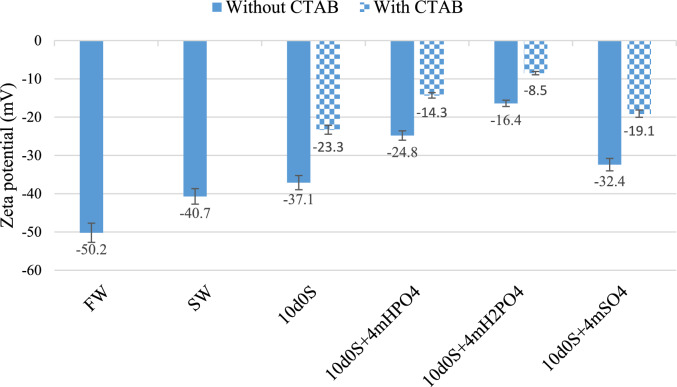
Zeta potential test results of smart water solutions in presence and absence of CTAB.

The zeta potential of clean carbonate rock powder at pH 8 is + 9.6 mV. After becoming oil-wet, the zeta potential changes to -54.9 mV^64^. The oil-wet rock powder was exposed to different solutions and zeta potential results in presence and absence of CTAB are shown in Fig. 12. This shift of zeta potential to more positive values demonstrate the ability of aqueous solutions to change wettability towards more water-wet condition. In 10d0S, it increases to -37.1 mV, for ${\text{SO}}_{4}^{2-}$, it increases to -32.4 mV; for ${\text{HPO}}_{4}^{2-}$, it increases to -24.8 mV; and for ${\text{H}}_{2}{\text{PO}}_{4}^{-}$, it increases to -16.4 mV. When negatively charged carboxyls are separated from the rock surface, the zeta potential becomes more positive, returning to its original value of + 9.6 mV.

The zeta potential of carbonate rock powder, consisting of almost 98% calcite, exposed to FW is -50.2 mV. High salinity increases the electric charge density around calcite, limiting ion movement and complex formation with carboxyl. Additionally, the high electric charge density around the calcite powder due to salinity further contributes to the high zeta potential. For the SW solution, the zeta potential increases more effectively than with the FW solution, reaching -40.7 mV. Reducing salinity and increasing ${\text{SO}}_{4}^{2-}$ concentration are key factors in changing zeta potential. Lower salinity decreases the electric charge density around the calcite powder, expanding the electric double layer and allowing ions to move more freely. This enables ${\text{SO}}_{4}^{2-}$ to approach the rock surface, allowing carboxyls to form ionic complexes with magnesium or calcium cations and move away from the rock surface. Consequently, the carbonate rock surface becomes more positive, and the zeta potential reaches -40.7 mV. The zeta potential of solution 10d0S is -37.1 mV, indicating that reducing salinity is more effective. Further reduction in salinity and expansion of the double layer improve ion mobility. The zeta potential of $10\text{d}0\text{S}+4{\text{mSO}}_{4}^{2-}$ shows that this reduction in salinity, along with increased sulfate anion concentration, makes the zeta potential more positive, reaching -32.4 mV. When 10d0S and $10\text{d}0\text{S}+4{\text{mSO}}_{4}^{2-}$ are compared, it is observed that the increase in ${\text{SO}}_{4}^{2-}$ concentration does not render the rock surface charge more negative than that of 10d0S; however, a positive effect on the zeta potential is noted, increasing it to -32.4 mV. This occurs because decreasing salinity reduces inert sodium and chlorine ion concentrations, while increasing ${\text{SO}}_{4}^{2-}$ anions promote the formation of carboxyl complexes with divalent cations.

Using the ${\text{HPO}}_{4}^{2-}$, which has a zeta potential of -24.8 mV, improves the separation of carboxyl from the rock surface. However, in the smart water solution containing ${\text{H}}_{2}{\text{PO}}_{4}^{-}$, the zeta potential is more positive (-16.4 mV) than the ${\text{HPO}}_{4}^{2-}$ (-24.8 mV) and ${\text{SO}}_{4}^{2-}$ (-32.4 mV) solutions. This difference occurs because ${\text{H}}_{2}{\text{PO}}_{4}^{-}$ releases H + ions when dissolved in water. These protons are highly reactive and form carboxylic acid with carboxyls on the calcite surface, which is soluble in water.

10 $$\left(\text{R}-\text{COO}-\right)+\left(\text{H}+\right)\stackrel{}{\to }\left(\text{R}-\text{COOH}\right)$$

In addition, ${\text{HPO}}_{4}^{2-}$ and ${\text{H}}_{2}{\text{PO}}_{4}^{-}$ anions in $10\text{d}0\text{S}+4{\text{mH}}_{2}{\text{PO}}_{4}^{-}$ smart water solution can also act as catalysts and calcium and magnesium cations can separate other carboxyls from the rock surface. Moreover, the zeta potential of $10\text{d}0\text{S}+4{\text{mH}}_{2}{\text{PO}}_{4}^{-}$ (-16.4 mV) is significantly lower than that of $10\text{d}0\text{S}+4{\text{mHPO}}_{4}^{2-}$ (-24.8 mV). This occurs because ${\text{H}}_{2}{\text{PO}}_{4}^{-}$ solutions have fewer negative charges than ${\text{HPO}}_{4}^{2-}$ solutions. Some ${\text{H}}_{2}{\text{PO}}_{4}^{-}$ anions release their hydrogen, becoming divalent anions. Consequently, rock powder treated with ${\text{H}}_{2}{\text{PO}}_{4}^{-}$ shows a lower zeta potential than rock powder treated with ${\text{HPO}}_{4}^{2-}$. FTIR results support this difference in negative charge density. Comparing FTIR spectra of smart water solutions containing ${\text{SO}}_{4}^{2-}$ and ${\text{HPO}}_{4}^{2-}$ shows that, after treating rocks with CTAB, peaks related to alkanes strengthen, while carboxyl groups are largely removed from the surface. This indicates CTAB absorption on the rock surface, driven by anion absorption and a reduction in positive charge around the rock.

In the presence of CTAB, smart water solutions enriched with anions show a more positive zeta potential. For ${\text{SO}}_{4}^{2-}$, it increased from -32.4 mV to -19.1 mV; for ${\text{HPO}}_{4}^{2-}$, it increased from -24.8 mV to -14.3 mV; and for ${\text{H}}_{2}{\text{PO}}_{4}^{-}$, it increased from -16.4 mV to -8.5 mV. This indicates that CTAB aids the process of wettability alteration. CTAB is more effective in reducing the negative electric charge and therefore the zeta potential increase in ${\text{SO}}_{4}^{2-}$ solution (-32.4 mV to -19.1 mV) compared to ${\text{HPO}}_{4}^{2-}$ (-24.8 mV to -14.3 mV) and ${\text{H}}_{2}{\text{PO}}_{4}^{-}$ (-16.4 mV to -8.5 mV). Anions in smart water solutions approach the rock surface, reducing positive charge density and increasing negative charge density. This allows calcium and magnesium cations to approach the surface, where they form complexes with negatively charged carboxyls, moving them away from the surface. Additionally, CTAB’s hydrocarbon chain and positive charge make it effective in forming complexes with carboxyls on the surface. This reduces rock surface carboxyls and shifts the zeta potential towards more positive values. The change in zeta potential for the ${\text{H}}_{2}{\text{PO}}_{4}^{-}$ solution is less than for the others. ${\text{H}}_{2}{\text{PO}}_{4}^{-}$ effectively removes carboxyls from the surface and has a lower electric charge than with ${\text{SO}}_{4}^{2-}$ and ${\text{HPO}}_{4}^{2-}$. On the other hand, the presence of the positively charged hydrogen ion also affects the zeta potential. Despite predictions that the hydrogen ion’s reactivity might marginalize CTAB’s performance, CTAB remains effective. It reduces oil-wetness and the contact angle between oil droplets and carbonate rock, resulting in a more positive zeta potential.

### IFT

**Fig. 13:**
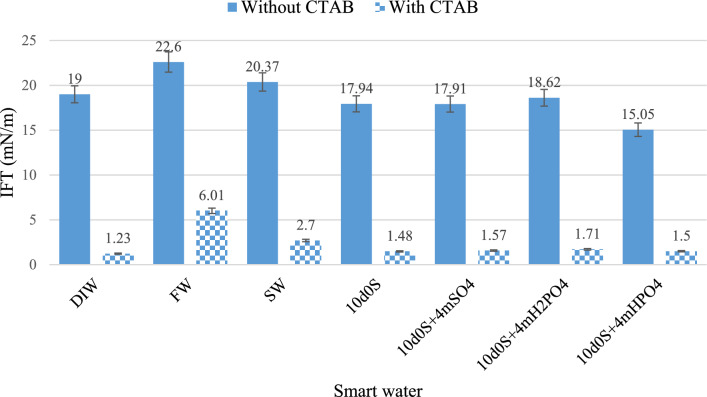
IFT results between crude oil and different smart waters in presence and absence of CTAB.

The results of IFT tests between the crude oil and different solutions in presence and absence of CTAB are represented in Fig. 13. The FW solution has the highest IFT with oil, due to greater salinity. Therefore, the salting-out mechanism and the small stern layer cause the high IFT^48,65^. These factors prevent ions from operating freely and do not provide space for large polar hydrocarbon molecules to enter. The SW solution has lower salinity than the FW solution. Additionally, it contains sulfate anions with higher concentration than FW. These anions reduce the IFT of salt water with oil (for FW, the IFT is 22.6 mN/m and in SW, it reaches 20.37 mN/m). They absorb polar oil molecules and form hydrogen bonds, which reduce the surface tension^78^. The DIW solution, which lacks salinity and ions, has the lowest surface tension (19 mN/m) compared to FW (22.6 mN/m) and SW (20.37 mN/m) solutions with oil. This indicates that the salinity of FW and SW solutions is still too high to achieve optimal IFT^79^. The IFT values from other smart water solutions (10d0S, $10\text{d}0\text{S}+4{\text{mSO}}_{4}^{2-}$, $10\text{d}0\text{S}+4{\text{mH}}_{2}{\text{PO}}_{4}^{-}$, $10\text{d}0\text{S}+4{\text{mHPO}}_{4}^{2-}$) with oil confirm that the water salinity and the presence of ions decrease IFT, but the salinity must be at an optimal level^48,65^.

The 4m times increase in the molar concentration of with ${\text{SO}}_{4}^{2-}$ in $10\text{d}0\text{S}+4{\text{mSO}}_{4}^{2-}$ compared to 10d0S shows that this anion helps reduce surface tension, even if only slightly (for 10d0S, the IFT is 17.94 mN/m and for $10\text{d}0\text{S}+4{\text{mSO}}_{4}^{2-}$, the IFT is 17.91 mN/m). Sulfate ions form ion micelles and polar hydrocarbons. They can also absorb into the acidic compounds in oil. Therefore, as a bivalent ion, sulfate forms more stable complexes^80^. On the other hand, In the $10\text{d}0\text{S}+4{\text{mH}}_{2}{\text{PO}}_{4}^{-}$ solution, the ${\text{H}}_{2}{\text{PO}}_{4}^{-}$ anion decreases the pH of the environment by releasing H + . Despite 4m times increase in the anion, the IFT value remains higher (18.61 mN/m) than in the $10\text{d}0\text{S}+4{\text{mSO}}_{4}^{2-}$(17.91 mN/m) and $10\text{d}0\text{S}+4{\text{mHPO}}_{4}^{2-}$ (15.05 mN/m) solutions. Moreover, the $10\text{d}0\text{S}+4{\text{mHPO}}_{4}^{2-}$ solution has a higher pH than the $10\text{d}0\text{S}+4{\text{mH}}_{2}{\text{PO}}_{4}^{-}$ and $10\text{d}0\text{S}+4{\text{mSO}}_{4}^{2-}$ solutions. This indicates that the oil contains more acidic compounds, such as carboxyls. At a higher pH, these compounds are more easily absorbed by divalent anions and enter the aqueous phase. This absorption reduces the IFT between the aqueous solution and oil.

As shown in Fig. 13, Combining smart water solutions with CTAB significantly reduces the surface tension between water and oil. This occurs because the hydrocarbon heads in CTAB molecules forms complexes with hydrocarbon compounds in the oil at the contact surface of the aqueous solution and the oil. However, based on the results, CTAB’s effect on reducing surface tension decreases as salinity increases. This happens because the presence of anions and cations in the aqueous solution and the stern layer near the surface hinders large CTAB molecules from accessing the oil. Consequently, fewer CTAB-oil hydrocarbon complexes form, resulting in higher surface tension. On the other hand, by examining the pH pattern of aqueous solutions, we can conclude that lower pH levels enhance CTAB’s effect on reducing surface tension. $10\text{d}0\text{S}+4{\text{mH}}_{2}{\text{PO}}_{4}^{-}$ has lower pH (4.5) than $10\text{d}0\text{S}+4{\text{mHPO}}_{4}^{2-}$ (7.4), but IFT reduction in $10\text{d}0\text{S}+4{\text{mH}}_{2}{\text{PO}}_{4}^{-}$ (from 18.62 mN/m to 1.71 mN/m) is more than $10\text{d}0\text{S}+4{\text{mHPO}}_{4}^{2-}$ (from 15.05 mN/m to 1.5 mN/m)**.**

### Spontaneous imbibition

**Fig. 14:**
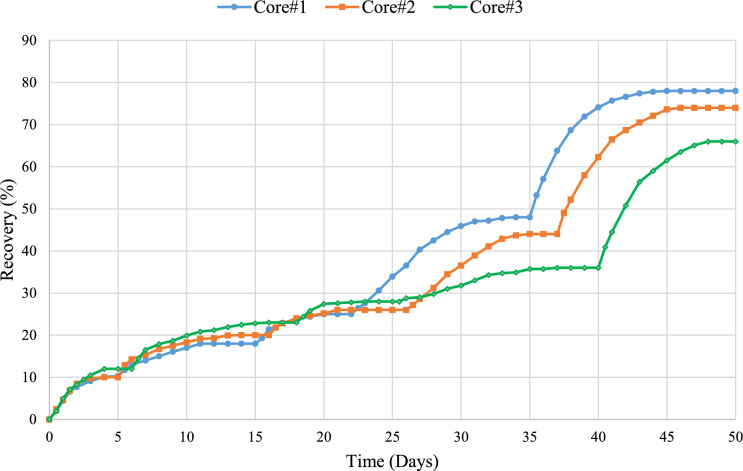
Results of oil recovery by spontaneous imbibition for all three cores. $10\text{d}0\text{S}+4{\text{mSO}}_{4}^{2-}$, $10\text{d}0\text{S}+4{\text{mHPO}}_{4}^{2-}$ and $10\text{d}0\text{S}+4{\text{mH}}_{2}{\text{PO}}_{4}^{-}$ solutions are used for cores #1, #2 and #3, respectively.

To assess the impact of ${\text{SO}}_{4}^{2-}$, ${\text{HPO}}_{4}^{2-}$ and ${\text{H}}_{2}{\text{PO}}_{4}^{-}$ ion solutions on oil production, spontaneous imbibition tests were conducted with and without CTAB. Figure 14 shows oil recovery under different brine waters. This test was conducted in five steps for each solution, using specific plugs for each one. To eliminate the effects of differences in the porous medium and ensure accurate comparisons, three plugs were used with nearly identical petrophysical characteristics. The test was carried out at 90°C using an Amott cell. The plugs were saturated with 80% oil and 20% FW. The plugs aged for almost 8 weeks, similar to the aging process of the rock sections. In steps 1 to 4, oil recovery was calculated using FW, SW, 10d0S, and smart water for each anion solution. In the last step, the effect of 1 CMC CTAB was investigated along with each anion solution on oil recovery. The imbibition process was continued in each brine water until oil production stopped. To assess the oil recovery of ${\text{H}}_{2}{\text{PO}}_{4}^{-}$, ${\text{HPO}}_{4}^{2-}$, and ${\text{SO}}_{4}^{2-}$ solutions, the first, second, and third cores were used respectively. Therefore, the values ​​reported below are for the first, second, and third cores, respectively.

In the first step, the plugs were imbibed in FW for 5, 5 and 6 days. The oil recovery values reached 10%, 10%, and 12%, respectively. In the second step, after oil production ceased in FW, the plugs were imbibed in SW. During 10, 11 and 12 days of imbibition in SW, the oil recovery amounts reached 18%, 20%, and 23%. In the third step, after oil production plateaued, the plugs were imbibed in 10d0S. Imbibition in the smart water continued for 7, 8, 7 days and oil recovery reached 25%, 26%, and 28%. In the fourth step, ${\text{H}}_{2}{\text{PO}}_{4}^{-}$, ${\text{HPO}}_{4}^{2-}$ and ${\text{SO}}_{4}^{2-}$ solutions were used for imbibition in the cores. During 12, 14, 15 days of imbibition in these solutions, oil recovery reached 48%, 44%, and 36%. In the final step, the solutions were used with CTAB. After introducing CTAB, the oil recovery curve slope rose sharply, reaching 78%, 74%, and 66%, respectively.

According to the IFT and imbibition tests, smart waters containing mono- and dihydrogen phosphate ions perform better than $10\text{d}0\text{S}+4{\text{mSO}}_{4}^{2-}$ in surface tension reduction. Figure 13 shows that different ions reduce surface tension almost similarly. Surface tension reduction becomes the predominant mechanism when IFT values are ultralow (less than 0.1 mN/m)^81,82^. Therefore, in this study, wettability alteration is the main mechanism for increasing oil recovery in the absence and presence of CTAB. Moreover, in presence of CTAB (Last stage of imbibition), the sharp increase in production is due to a significant reduction in IFT. Consequently, the results of this experiment confirm the findings of previous tests, showing that oil production in ${\text{H}}_{2}{\text{PO}}_{4}^{-}$ is higher compared to ${\text{HPO}}_{4}^{2-}$ and ${\text{SO}}_{4}^{2-}$.

## Conclusions

This study included a comprehensive array of tests: contact angle measurements, FTIR-ATR imaging, zeta potential measurements, and spontaneous imbibition analyses. These tests dissected the mechanisms of smart waters enriched with three distinct anions, ${\text{H}}_{2}{\text{PO}}_{4}^{-}$, ${\text{HPO}}_{4}^{2-}$ and ${\text{SO}}_{4}^{2-}$, both with and without CTAB. Their efficacy in altering the wettability of oil-wet carbonate rock and in reducing the surface tension with crude oil was examined. To investigate the impact of anions, their molar concentrations were varied to 1/3, 1, 2, 4, and 8 times the sulfate concentration present in the 10d solution. These anions were introduced into a base smart water solution, defined as 10 times diluted SW without sulfate anions (10d0S). The results led to the following conclusions:

- The contact angle test demonstrated that the wettability alteration achieved with ${\text{H}}_{2}{\text{PO}}_{4}^{-}$ and ${\text{HPO}}_{4}^{2-}$ smart waters surpassed that of ${\text{SO}}_{4}^{2-}$.
- Increasing anion concentration to an optimal value (4 times the sulfate concentration in 10d) enhanced wettability alteration.
- A consistent concentration of CTAB changed the wettability to a more water-wet state on the rock surface.
- Ion exchange and surface dissolution mechanisms increased wettability alteration.
- FTIR-ATR analysis revealed no CTAB adsorption onto the rock surface with the $10\text{d}0\text{S}+4{\text{mH}}_{2}{\text{PO}}_{4}^{-}$ smart water solution in the presence of surfactant. However, limited CTAB adsorption occurred with $10\text{d}0\text{S}+4{\text{mSO}}_{4}^{2-}$ smart water due to the higher negative charge density of anions around the rock surface.
- ${\text{H}}_{2}{\text{PO}}_{4}^{-}$ smart water significantly increased the zeta potential of oil-wet rock powder, especially with CTAB. ${\text{H}}_{2}{\text{PO}}_{4}^{-}$ effectively removed polar oil constituents from the rock surface, promoting a transition to a water-wet state.
- Incorporating CTAB into smart water solutions significantly decreased the IFT, with minimal discrepancy between anions, less than 1 mN/m.
- Wettability alteration was the primary mechanism for enhancing oil recovery.
- The ${\text{H}}_{2}{\text{PO}}_{4}^{-}$ solution exhibited the highest oil recovery both with and without surfactant.

While the selection of the 4m anion concentration in this study was based on experimental data, further investigation could incorporate numerical modeling and surface chemistry simulations to predict optimal ion concentrations

## Data Availability

All data that supports the findings of this study are available from the corresponding author upon reasonable request.
